# The association of cesarean section with overweight and neurodevelopment of Chinese children aged 1–5 months

**DOI:** 10.3389/fped.2022.940422

**Published:** 2022-08-23

**Authors:** Xiaoguo Zheng, Ruili Li, Lihong Wang, Huimin Yang, Linlin Li, Jiayin Cui, Wenhua Zhao, Zhenyu Yang, Qian Zhang, Tao Xu, Yuying Wang, Bowen Chen

**Affiliations:** ^1^Department of Children Health and Development, Capital Institute of Pediatrics, Beijing, China; ^2^National Institute for Nutrition and Health, Chinese Center for Disease Control and Prevention, Beijing, China; ^3^National Center for Women and Children’s Health, Chinese Center for Disease Control and Prevention, Beijing, China

**Keywords:** cesarean section, mode of delivery, neurodevelopment, development, China

## Abstract

**Objective:**

The purpose of this survey was to explore the association of delivery mode with overweight and neurodevelopment of Chinese infants aged 1–5 months.

**Materials and methods:**

This study was based on a cross-sectional survey. Data for this study were obtained from the Children’s Nutrition and Health System Survey in China which was conducted from 2019 to 2020. Characteristics of parents and children and the delivery mode were obtained using interview-administered questionnaires. Body mass index-for age z-score (BMI z) was calculated using World Health Organization (WHO) child growth standards. Children’s neurodevelopment was assessed by a trained child health care physician using the Child Psychological Development Scale. The association of delivery mode with infant overweight was analyzed using a multivariable logistic regression model. We conducted a multivariable linear regression model to explore the relationship between delivery modes with neurodevelopment.

**Results:**

In total, the present analysis included 1,347 children aged 1–5 months, 35.61% were born *via* cesarean section, of which 15.21% were overweight. After adjustment for infant characteristics and parental factors, the cesarean section was significantly related with the likehood of being overweight [*OR* = 1.95; 95% confidence interval (CI): 1.27 to 2.98]. Children born *via* cesarean section had a 3.41-point decrease in gross motor development (β = −3.41; 95% CI: −5.77 to −1.05), a 3.65-point decrease in fine motor development (β = −3.65; 95% CI: −6.03 to −1.28), and a 2.96-point in language development (β = −2.96; 95% CI: −5.20 to −0.73), a 1.65-point in total development (β = −1.65; 95% CI: −3.17 to −0.14) compared with those who were vaginal birth.

**Conclusion:**

In our study population, cesarean section was associated with overweight and neurodevelopment outcomes. The cesarean section might increase the likehood of infant overweight, and might decrease the developmental scores of gross motor, fine motor and language. Further studies should be conducted to verify the associations and explore the possible mechanisms.

## Introduction

The rising rate of cesarean section has become a social public issue worldwide. Originally, the cesarean section was a life-saving surgery for women and newborns when complications occurred. Over time, the cesarean section rate in both developed and developing countries has increased rapidly. During the past three decades, global cesarean section rates have increased from approximately 6% in 1990 to 21% in 2015 (1), well above the 10–15% recommended by the WHO (2). In the United States, the cesarean section rate has increased from 27% in 1997 to 31.8% in 2011, the rate of cesarean section has increased from 18% in 1997 to 25% in 2010 in the United Kingdom, while in Iran, the cesarean section rate is almost 40% (3). Nevertheless, the proportion of cesarean section have risen from 18% in 1990–1992 to 34.9% in 2014 and was over 50% in some major cities of China (4). One study conducted in China from 2013 to 2016 showed 20% of cesarean section might be non-medically necessary (5). Previous research has found that cesarean section increases the risk of a variety of diseases in the offspring, including allergies (6), type 1 diabetes (7), overweight/obesity (8, 9) and may be linked to poor child cognitive outcome (10) and lower academic performance (11). Some studies have discussed the biological mechanism hypothesis of the relationship between cesarean section and the negative health of the offspring. The cesarean section may interfere sensory activation and immune due to be short of stress response, affect immune system development, modifying epigenetic regulation in DNA methylation, or disrupting bacterial colonization (10, 12).

The cesarean section was linked with childhood overweight and excess weight gain in the first 1,000 days (13, 14). The United States infants born by cesarean section gained more weight than those delivered vaginally (15). Compared to vaginal delivery, the risk of being overweight was 2.44 times higher for 1 year old infants born by elective cesarean section in an Indian study (14). Also, one Chinese cohort study showed that the risk of being overweight and obese increased by 24 and 29%, respectively, in preschool children delivered by cesarean section (8). However, few studies had reported that cesarean section was not significantly associated with overweight/obesity in children (16, 17). Whether cesarean section is associated with children’s development, the existing research is inconsistent. Previous studies have found that pre-labor cesarean section may be associated with school performance (11), and poor child cognitive score outcomes (10) and a higher incidence of autism spectrum disorders (18). Zaigham M et al. reported that the infants born by pre-labor cesarean section had obviously lower assessment scores in all developmental domains at the 4-month assessment and lower score in the gross-motor skills domain at the 12-month evaluation compared to vaginally born infants (19). But other studies did not support a strong link between cesarean section and neurodevelopment or poorer health in children (20, 21).

Given the inconsistencies of the findings, the conflicting results can be partly interpreted by different adjustment confounding factors, mainly the children’s age when the assessment was conducted. In China, few studies have explored the relationship between cesarean section and overweight in childhood; however, study sample didn’t focus on infants’ overweight. So, the hypothesis of this study was that there was a link between cesarean section and the infant’s overweight and neurodevelopment.

## Materials and methods

### Participants

This study was based on a cross-sectional survey. The data were extracted from the Children’s Nutrition and Health System Survey in China. The sampling method in this study has been detailed previously (22). In short, stratified multi-stage cluster sampling was used to select participants in each province. This study included children aged 1 to 5 months and the data were selected from nine provinces: Zhejiang (*n* = 156), Beijing (*n* = 143), Jilin (*n* = 148), Liaoning (*n* = 146), Shanxi (*n* = 152), Jiangxi (*n* = 146), Hunan (*n* = 164), Qinghai (*n* = 148), and Yunnan (*n* = 144). The survey was conducted in 2019–2020. The inclusion criteria were (1) healthy children who had lived locally at the survey site for more than 6 months; (2) birth weight ≥ 2,500 g; (3) gestational age ≥ 37 weeks; (4) not a twin or multiple births; and (5) having no serious illnesses or chronic health problems.

### Anthropometrics

The infants’ weight and height were measured by well-trained staff from local Centers for Disease Control and Prevention and community health center according to standard procedures (23). The weight was measured on a lever scale to an accuracy of 0.1 kg. Length was measured using a pediatric length board to an accuracy of 0.1 cm with the infants in a recumbent position. The age-and sex-specific BMI z-scores (BMI z) was calculated according to the WHO Child Growth Standards for infants aged 0–60 months. Overweight of infants was categorized by BMI *z* ≥ 85th percentile.

### Variables

Characteristics of parents, children, and the mode of delivery were obtained using interview-administered questionnaires. The mode of delivery was divided into cesarean section and vaginal delivery. Parental characteristics included maternal age (“< 35 years” or “≥ 35 years”), maternal education (“Bachelor and above” or “College” or “High school” or “Middle school and below”), gestational weight gain (continuous variable), paternal age (“< 35 years” or “≥ 35 years”), paternal education (“Bachelor and above” or “College” or “High school” or “Middle school and below”), and monthly household income per capita (“1,501–3,000 RMB” or “3,001–5,000 RMB” or “≥ 5,000 RMB”). The child characteristics comprised sex (“Male” or “Female”), birth weight (“Formal” or “Macrosomia”), feeding methods (“Breast feeding” or “Formula feeding” or “Mixed feeding”), residential area (“Urban” or “Rural”), and parity (“Primipara” or “Multipara”).

### Assessment of neurodevelopment

The children’s neurodevelopment was assessed by a standardized trained child health care physician using the Child Psychological Development Scale (WS/T 580-2017). The scale is an effective, reliable, and accredited tool, developed by the Capital Institute of Pediatrics in China. This scale is used to assess the neurodevelopmental level of children aged 0–6 years and has been widely used in pediatric and maternal and child health care institutions in China since 1984. The scale is consisted of five domains (gross motor, fine motor, adaptability, language, and social behavior). And the developmental quotient (DQ, *DQ* = mental age÷actual age in months × 100) is used to assess children’s total neurodevelopmental level and five domains [gross motor development quotient (GMDQ), fine motor development quotient (FMDQ), adaptability developmental quotient (ADQ), language development quotient (LDQ), and social behavior developmental quotient (SBDQ)].

### Statistical analysis

We used SAS for Windows, Version 9.2 (SAS Institute Inc., Cary, NC, United States) to conduct all the analyses in this study. Characteristics of the children and their parents were analyzed using *t* test for continuous measures or χ2 tests of independence (for categorical variables). Comparisons of DQs scores in different domains in cesarean section and vaginal delivery children were conducted using a *t*-test. The association of infant overweight with the delivery mode was examined using multivariable logistic regression, and we conducted multivariable linear regression models to assess the associations between DQ scores (total scale and five domains) and delivery models (cesarean section and vaginal delivery) after adjusting for several confounders (birth weight, maternal age, paternal age, gestational weight gain, child sex, delivery mode, residential area, maternal education, paternal education, parity, feeding methods, and monthly household income per capita). In all the statistical tests, *P* < 0.05 was considered statistical significance.

## Results

### Characteristics of the participants

As shown in Table 1, our study included a total of 1,347 children aged 1–5 months. Approximately 35.63% of children (*n* = 480) were born by cesarean section and 64.37% of children (*n* = 867) were vaginal delivery. The mean maternal age was 29.97 years. Mothers who were 35 years or older, with a higher education level, who lived in an urban area, were primipara, and gained more weight during pregnancy were tend to deliver their children *via* cesarean section (*p* < 0.05). Out of 480 children born *via* cesarean section, the BMI z was 0.18 (SD: 1.38), of 11.25% were macrosomia and 21.67% were formula fed, and 16.61% of the vaginal delivery children were formula fed. Children’s sex, paternal education level, and monthly household income per capita were not significantly related with delivery mode (*p* > 0.05).

**TABLE 1:** Characteristics of the study population by delivery mode (*n* = 1,347).

		Cesarean section (*n* = 480)	Vaginal delivery (*n* = 867)	*P*
Birth weight (kg), mean (SD)	1347	3.42 (0.44)	3.27 (0.36)	< 0.001
Maternal age (years), mean (SD)	1347	30.67 (4.83)	29.26 (4.37)	< 0.001
Paternal age (years), mean (SD)	1347	32.40 (5.08)	31.24 (4.88)	< 0.001
GWG (kg), mean (SD)	1347	14.41 (4.63)	13.30 (4.10)	< 0.001
Child BMI z, mean (SD)	1347	0.18 (1.38)	−0.09 (1.30)	0.025
Child sex, n (%)				0.687
Male	672	243 (50.63)	429 (49.48)	
Female	675	237 (49.37)	438 (50.52)	
Birth weight, n (%)				< 0.001
Formal	1257	426 (88.75)	831 (95.85)	
Macrosomia	90	54 (11.25)	36 (4.15)	
Feeding methods, n (%)				0.011
Breast feeding	142	59 (12.29)	83 (9.57)	
Formula feeding	248	104 (21.67)	144 (16.61)	
Mixed feeding	957	317 (66.04)	640 (73.82)	
Residential area, n (%)				0.006
Urban	829	319 (66.46)	510 (58.82)	
Rural	518	161 (33.54)	357 (41.18)	
Maternal age, n (%)				< 0.001
<35 years	1167	392 (81.67)	775 (89.39)	
≥ 35 years	180	88 (18.33)	92 (10.61)	
Paternal age, n (%)				< 0.001
<35 years	1029	334 (69.58)	695 (80.16)	
≥ 35 years	318	146 (30.42)	172 (19.84)	
Maternal education, n (%)				< 0.001
Middle school and below	290	136 (28.33)	154 (17.76)	
High school	233	88 (18.33)	145 (16.72)	
College	418	114 (23.75)	304 (35.06)	
Bachelor and above	406	142 (29.58)	264 (30.45)	
Paternal education, n (%)				0.082
Middle school and below	259	101 (21.04)	158 (18.22)	
High school	231	92 (19.17)	139 (16.03)	
College	459	144 (30.00)	315 (36.33)	
Bachelor and above	398	143 (29.79)	255 (29.41)	
Parity, n (%)				< 0.001
Multipara	512	211 (43.96)	301 (34.72)	
Primipara	816	252 (56.04)	564 (29.53)	
Monthly household income per capita, n (%)				0.084
1,501–3,000 RMB	91	42 (8.75)	49 (5.65)	
3,001–5,000 RMB	547	195 (40.63)	352 (40.60)	
≥ 5,000 RMB	709	243 (50.63)	466 (53.75)	

*P*-values obtained from χ2 tests of independence (for categorical variables) or one-way ANOVA (for continuous measures). GWG, gestational weight gain.

### Results of cesarean section and infant overweight

The result of the logistic regression model was shown in Table 2. Among infants delivered by cesarean section, the percentage of overweight was 15.21%, and it was higher than the overweight rate (13.03%) in the vaginal delivery group. Compared to vaginal born, infants born by cesarean section had 1.28 times the likehood of being overweight before adjusting for covariates (95% CI: 1.07–1.56). After adjustment for infant characteristics and parental factors, the cesarean section was significantly related with the likehood of being overweight (*OR* = 1.95; 95% CI: 1.27 to 2.98) compared to those vaginal born.

**TABLE 2:** Logistic regression associated of delivery mode with infant overweight.

Mode of delivery	No. of overweight infants (%)	Unadjusted OR (95% CI)	Adjusted OR (95% CI)
Vaginal delivery	113 (13.03)	1.0	1.0
Cesarean section	73 (15.21)	1.28 (1.07 to 1.56)	1.95 (1.27 to 2.98)

Adjusted for maternal age, paternal age, maternal education, paternal education, monthly household income per capita, gestational weight gain, birth weight, residential area, and feeding methods.

### Compare of the total and five subscale scores between cesarean section and vaginal delivery groups

**FIGURE 1:**
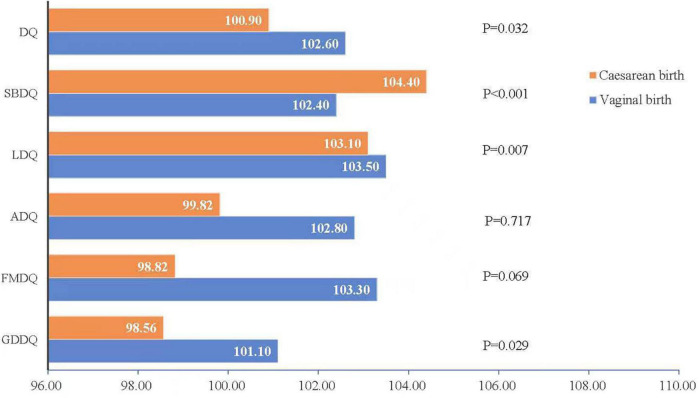
Compare of the total and five subscale scores between cesarean section and vaginal delivery.

Figure 1 presents the comparison of the total and five subscale scores between cesarean and vaginal birth groups. There was a statistical significance in the scores of GDDQ, FMDQ, ADQ, and DQ between vaginal delivery and cesarean section groups (*P* < 0.05). The children in the cesarean section group had lower scores in GDDQ (98.56 ± 21.54), FMDQ (98.82 ± 20.49), ADQ (99.82 ± 19.58), and DQ (100.90 ± 13.31) than the vaginal delivery group [GDDQ (101.10 ± 19.74), FMDQ (103.30 ± 20.17), ADQ (102.80 ± 18.87), and DQ (102.60 ± 13.17)]. There was no significant difference in language and social behavior development domains (*P* > 0.05).

### Results of cesarean section and infant neurodevelopment

The results of the multivariable linear regression association of delivery mode with infant neurodevelopmental outcomes were shown in Table 3. After adjusting for potential confounders (maternal education, maternal age, paternal education, paternal age, monthly household income per capita, parity, gestational weight gain, birth weight, residential area, and feeding methods), children who were born by cesarean section had a 3.41-point decrease in gross motor development (β = −3.41; 95% CI: −5.77 to −1.05), a 3.65-point decrease in fine motor development (β = −3.65; 95% CI: −6.03 to −1.28), and a 2.96-point in language development (β = −2.96; 95% CI: −5.20 to −0.73), a 1.65-point in total development (β = −1.65; 95% CI: −3.17 to −0.14) compared with those who were vaginal delivery.

**TABLE 3:** Multivariable linear regression association of delivery mode with infant neurodevelopmental outcomes (*n* = 1,347).

	GDDQ			FMDQ			ADQ			LDQ			SBDQ			DQ		
	β	95%CI	*P*	β	95%CI	*P*	β	95%CI	*P*	β	95%CI	*P*	β	95%CI	*P*	β	95%CI	*P*
Vaginal delivery	1.0			1.0			1.0			1.0			1.0			1.0		
Cesarean section	−3.41	−5.77 to −1.05	0.005	−3.65	−6.03 to −1.28	0.003	−0.33	−2.61 to 1.95	0.778	−2.96	−5.20 to −0.73	0.010	2.09	−0.23 to 4.40	0.249	−1.65	−3.17 to −0.14	0.033

Adjusted for maternal age, paternal age, maternal education, paternal education, monthly household income per capita, gestational weight gain, birth weight, residential area, and feeding methods.

## Discussion

Our study shows that cesarean section was associated with overweight and neurodevelopment outcome of children aged 1 to 5 months. The cesarean section might increase the likehood of infant overweight, and might decrease the developmental scores of gross motor, fine motor, and language. In our study, the multivariable logistic regression model showed cesarean section was obviously related with the likehood of being overweight. It was in line with a cohort study in Copenhagen which reported that infants delivered by cesarean section had a higher mean BMI z when the infants aged 6 months compared to that vaginal delivery (24). A systematic review reported that cesarean section had a RR of 1.34 for childhood obesity when compared to vaginal delivery across ages 2–18 years (25). A cohort study of 9,103 children in China reported a 29% increased risk of being obesity in children aged 3–6 years who were delivered by cesarean section (8). In the same way, Pei et al. disclosed a distinctly relationship between cesarean section and obesity in 1,734 German children aged 2 years (26) and a Peruvian prospective cohort study also found obviously associations between cesarean section and the risk of obesity (RR: 2.25; 95% CI: 1.36–3.74) and overweight (RR: 1.51; 95% CI: 0.98–2.35) in children aged 5 years (27). Other studies have different conclusion, they classified cesarean section as emergency cesarean section or elective cesarean section, and found elective cesarean section was associated with a higher risk of infant overweight, but emergency cesarean section was not (14, 28). In this study, the cesarean section didn’t differentiate elective cesarean section and emergency cesarean section, which may have some confounding effect on the results. Two other studies found no significant relationship that the cesarean section type varied the risk of being obesity and overweight in children aged 2–5 years (16, 17).

Previous studies showed that cesarean section was linked to poor child cognitive outcomes (10) and school performance (11). Our study reported that it was statistical significance in the scores of DQ, GDDQ, FMDQ, and ADQ between cesarean section and vaginal delivery groups (*P* < 0.05). The result was consistent with the result of Zaigham M et al., who disclosed that infants born by cesarean section (*n* = 66) had distinctly lower assessment scores (the validated Ages and Stages Questionnaire-II) in all developmental domains (communication, gross motor, fine motor, problem solving, and personal-social) at the 4 month assessment, and lower score in the gross-motor skills domain at the 12-month evaluation than infants delivered *via* vaginal delivery (*n* = 352) (19). Polidano et al. showed that children born by cesarean section performed a tenth of a standard deviation below in the national test scores for numeracy as compared to vaginally born children in 3,666 Australian children aged 4 to 9 years (10). According to a previous systematic review, the children born by cesarean section had an increased risk of attention-deficit/hyperactivity disorder and autism spectrum disorder when compared to vaginal delivery (29). An epidemiological study conducted in Norway, Sweden, Denmark, Finland, and Australia included 671,464 children delivered by cesarean section, reported the overall adjusted OR was 1.26 in cesarean section children compared with vaginal delivery (30). Other research, however, did not support the link between cesarean section and poor neurodevelopment in children (20, 21). Previous studies reported that maternal obesity is related to an excess risk of cesarean delivery and children overweight (31, 32). Also children born to mothers with gestational diabetes, which is linked with maternal obesity, are at higher risk for lower neurodevelopment and behavioral problems (33, 34). However, we did not consider maternal BMI and gestational diabetes in this study, which may have some confounding effect on the results.

There are several possible mechanisms to explain the relationship of delivery mode and child overweight and neurodevelopment. First, the gut microbiota composition was different by mode of delivery (35–37). The gut microbiota of children born by cesarean section was seeded from the mother’s skin and the hospital environment because they were not exposed to the mother’s birth canal. Hence, the infant’s gut microbiota differs from that of vaginal births (38) and this difference persists into adolescence or early adulthood (39). Second, microbiota play a crucial role in children’s overweight and brain development. Alternations in the microbiome of infants lead to changes in metabolic pathways of the infants (40), and the different postnatal development of the immune system was contributed by the differences in microbiota (41). Collins et al. found that gut microbiota can influence the brain development and behavior through communicating with the brain *via* the gut–brain axis (42). Third, the alternation of mother-child interactions was associated with the cesarean section (43) and the cesarean section was linked with lower rates of infants’ breastfeeding (44). Studies have reported that delivery by cesarean section may increase the risk of posttraumatic stress compare to vaginal delivery (45, 46). Mothers with cesarean section were more susceptible to have postpartum complications, which might affect mother-infant attachment and the infants’ neurodevelopment. After a cesarean section, the mothers might be more likely to have pain or sickness, which can affect the mothers to initiate and maintain the breastfeeding, or even lead to a reduction in breast milk production (47, 48). Lastly, cesarean section as an “artificial labor” might lead to epigenetic modifications of gene expression, and DNA methylation may be altered in certain cases (12).

On account of the cesarean section has some consequences for a child’s health and development, as well as subsequent pregnancies, the WHO recommends that cesarean section should be conducted based on the medical indications. In China, one cross-section survey reported that the national average cesarean section rate is 34.9% (49). In our study, the cesarean section rate was 35.6%, what is not clear of this study is whether all the cesarean sections were performed on medical necessary. The appropriate cesarean section rate in China needs to be discovered in the future studies.

## Strengths and limitations

There are several strengths in the present study. First, we used a multistage, stratified, randomized sampling method in this study, so study participants were a good representation of the general population of Chinese children. Second, participants were healthy, full-term infants without pregnancy complications or serious illness; therefore, this study excluded the potential confounding effect of various diseases. Third, the Child Psychological Development Scale (WS/T 580-2017) was used to examine developmental outcomes in our study. The developmental milestones of the children were fully or partially covered in this scale. This is a local scale in China, and the items are very suitable for Chinese children. There are also some limitations in this study. First, this study was cross-sectional designed; thus, the obvious associations found in this study were correlational relationships. The effects of cesarean section on child health outcomes should be conducted in a future study using a longitudinal study design. Second, in our study, the cesarean section didn’t differentiate elective cesarean section and emergency cesarean section, and maternal BMI and gestational diabetes were not accounted for in the present study owing to the limitations of data collection, which may have had some confounding effect on the results. Third, we didn’t include all the risk factors, such as genetic and familial risk factors which may also explain the relationship and these factors will be taken into account in the future studies.

## Conclusion

In our study population, cesarean section was associated with overweight and neurodevelopment outcomes. The cesarean section might increase the likehood of infants’ overweight and might decrease the developmental scores of gross motor, fine motor, and language. Further studies should be conducted to verify the associations and explore the possible mechanisms.

## Data availability statement

The raw data supporting the conclusions of this article will be made available by the authors, without undue reservation.

## Ethics statement

The studies involving human participants were reviewed and approved by the Ethics Committee on Human Research at the National Institute for Nutrition and Health, Chinese Center for Disease Control and Prevention (No. 2019-009). Written informed consent to participate in this study was provided by the participants or their legal guardian/next of kin.

## Author contributions

XZ contributed to the performance of the study, analyzed the data, and drafted the manuscript. RL, LW, HY, LL, and JC were responsible for the data collection and reviewed drafts of the manuscript. WZ contributed to the project administration. ZY, QZ, TX, and YW contributed to the methodology, investigation, and supervision. BC contributed to the writing – review and editing. All authors participated in the conceptualization process of review and revision, read, and agreed to the final manuscript.
